# Lab-on-a-Chip Systems for Aptamer-Based Biosensing

**DOI:** 10.3390/mi11020220

**Published:** 2020-02-20

**Authors:** Niazul I. Khan, Edward Song

**Affiliations:** 1 Department of Electrical and Computer Engineering, University of New Hampshire, Durham, NH 03824, USA; mk1125@wildcats.unh.edu; 2 Materials Science Program, University of New Hampshire, Durham, NH 03824, USA

**Keywords:** Lab-on-a-chip, point-of-care, portability, miniaturization, microfluidics, soft-lithography

## Abstract

Aptamers are oligonucleotides or peptides that are selected from a pool of random sequences that exhibit high affinity toward a specific biomolecular species of interest. Therefore, they are ideal for use as recognition elements and ligands for binding to the target. In recent years, aptamers have gained a great deal of attention in the field of biosensing as the next-generation target receptors that could potentially replace the functions of antibodies. Consequently, it is increasingly becoming popular to integrate aptamers into a variety of sensing platforms to enhance specificity and selectivity in analyte detection. Simultaneously, as the fields of lab-on-a-chip (LOC) technology, point-of-care (POC) diagnostics, and personal medicine become topics of great interest, integration of such aptamer-based sensors with LOC devices are showing promising results as evidenced by the recent growth of literature in this area. The focus of this review article is to highlight the recent progress in aptamer-based biosensor development with emphasis on the integration between aptamers and the various forms of LOC devices including microfluidic chips and paper-based microfluidics. As aptamers are extremely versatile in terms of their utilization in different detection principles, a broad range of techniques are covered including electrochemical, optical, colorimetric, and gravimetric sensing as well as surface acoustics waves and transistor-based detection.

## 1. Introduction

**Figure 1:**
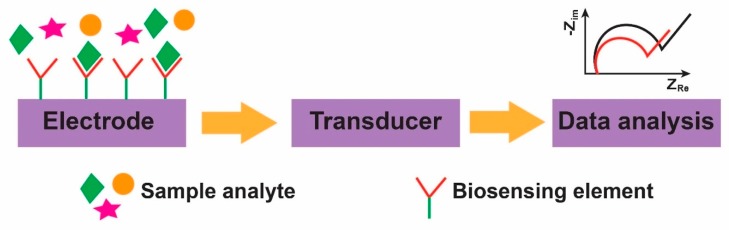
Schematic showing the operating principle of a biosensor. The plot gives a general description of the sensing mechanism.

According to the IUPAC (International Union of Pure and Applied Chemistry) definition, a biosensor is “a self-contained integrated device which is capable of providing specific quantitative or semi-quantitative analytical instrumentation using a biological recognition element (biochemical receptor) which is in direct spatial contact with a transducer element [1].” In general, a biosensor can be broadly defined as a device that converts a physical, chemical or biological event into a measurable signal. As can be seen in Figure 1, it consists of three main parts: (1) a biosensing element (aptamers, tissue, microorganism, organelles, cell receptors, enzymes, antibodies, proteins, etc.) which is a biologically derived material or biomimetic component that provides selectivity to the target analyte, (2) a transducer (physicochemical, optical, piezoelectric, electrochemical, etc.) that converts the resulting signal from the interaction of the analyte to the biosensing element into a measurable and quantifiable signal (in most cases electrical signal), and (3) the associated electronics or data analysis system which is primarily responsible for signal processing and user-friendly visualization of the sensing results. 

Aptamers have gained popularity in recent years as a target recognition element in sensing. First reported in 1990, aptamers have found applications in the fields of environmental and food safety and analytics as well as therapeutics and diagnostics in the healthcare sector [2,3,4]. Aptamers are typically DNA- or RNA-based oligonucleotide sequences which are designed to bind specifically to the target molecules. Aptamers are commonly selected through a process known as systematic evolution of ligands by exponential enrichment (SELEX) which is an iterative process to identify high affinity aptamer sequences from a large pool (10^15^–10^18^) of randomly generated oligonucleotide sequences. The first iteration begins with incubation of the target bioanalyte with a library of randomized oligonucleotide sequences in order to screen high-affinity sequences. The unbound aptamer sequences are washed away, and the bound aptamers are collected and amplified through polymerase chain reaction (PCR) to regenerate the oligonucleotide library for the next iteration of SELEX [5]. 

Various types of aptamer have been developed to recognize target species ranging from small ions to large proteins with high affinity and specificity. Furthermore, when compared with other biorecognition elements such as antibodies and enzymes, aptamers possess high chemical stability, mass-producibility and reusability, long shelf-life, low-cost and small size. Because of these advantages, aptamers are often used as an integral part of biosensors leading to the creation of the term *aptasensors* to describe aptamer-based biosensors. There are different classifications of aptasensors depending on the type of transduction mechanisms employed such as mass-based (i.e., quartz crystal microbalance (QCM)) [6,7], electrochemical (amperometric, voltammetry, impedimetric) [8,9,10,11,12,13,14,15,16,17,18], optical [19,20,21,22,23] or field-effect transistor (FET)-based methods [24,25,26].

The integration of aptasensors with microfluidics offers promising solutions for addressing some pressing healthcare challenges. Alternatively known as lab-on-a-chip (LOC) technology or miniaturized total analysis system (µTAS), microfluidics has versatile advantages to offer in biosensing including reduced sample volume and detection time, improved sensitivity due to high surface to volume ratio, high throughput by parallel operation, portability, and disposability. In addition, microfluidics-based biosensors enable real-time detection and an automated measurement process. This article reviews recent developments (last 5 years) on LOC systems for aptamer-based biosensing. We refer the readers to other previously published review articles that cover materials exclusively on either aptamer-based biosensing [27] or microfluidics-based biosensing [28,29,30,31,32,33,34]. Furthermore, although a LOC system typically comprises many analysis components such as sample collection, separation, filtration, mixing, and detection to name a few, as aptamers are used primarily as receptors for target biomolecules, this article will focus on the sensing component of the LOC that utilizes aptamers.

## 2. Microfluidics versus Macrofluidics

Microfluidics is the manipulation of fluid in submillimeter length scale. Due to the small dimension of microfluidic channels, fluidic behavior deviates from the macrofluidic behavior. Some interesting and often unintuitive properties may appear on this minute scale. For example, in microscale, diffusive mass transport dominates over convective mass transport. This is indicated by the Sherwood number *S_h_* which represents the ratio of the convective mass transfer to the diffusive mass transfer of the system and is defined as [35]: $S_{h}=\frac{kd}{D}$, where, *k* is the mass transport coefficient, *d* is the characteristic diameter of the channel and *D* is the diffusion coefficient. For macroscale systems, *S_h_* is large which indicates that convective transport is dominant over diffusive transport. By contrast, in the microfluidic system *S_h_* is much smaller due to the small channel geometry *d*, and consequently diffusive transport dominates the convective mass transport. This diffusive effect can be used in the passive mixing of two or more fluids as well as in the separation of particles based on the size in the microfluidic channel [36].

Laminar flow is another characteristic property of microfluidic systems. This is indicated by the Reynolds number (*R_e_*), which is defined as [37]: $R_{e}=\frac{\rho vd}{\eta }$, where *ρ* is the mass density of the fluid, *υ* is the fluid velocity, and *η* is the dynamic viscosity of the fluid. Due to small geometric dimensions (small *d*) of microfluidic systems, the Reynolds number *R_e_* can be as low as 1, which means the flow is dominated by viscous forces, and the flow is considered laminar. A consequence of this flow type is that two or more layers of fluid can flow side-by-side without any mixing other than by diffusive transport of their constituent molecular and particulate components [36]. 

Another significant property that distinguishes microscale systems from macroscale systems is the Bond number (*B_o_*). It is the ratio of the body forces (e.g. gravity) to the surface tension forces, and is defined as [35]: $B_{o}=\frac{\Delta \rho \alpha d^{2}}{\lambda }$, where Δ*ρ* is the density difference of the two phases across the interface, α is the acceleration associated with the body force, and λ is the surface tension between the two fluid phases. For microscale systems, small *d* results in a very low Bond number, which indicates the dominance of surface tension forces over body forces.

## 3. Different Types of Microfluidic Aptasensors

### 3.1. Microfluidic Aptasensors Based on Electrochemical Detection 

Electrochemical biosensors provide a convenient tool for quantifying the analyte due to its direct conversion of a chemical reaction into an electrical signal. There are several classifications of electrochemical sensing such as Faradaic current-based sensing (amperometric/voltammetric), potential or charge accumulation-based sensing (potentiometric), or electrical conductivity-based sensing (conductometric). Electrochemical impedance spectroscopy (EIS), or impedimetric sensing, is also a commonly used technique where a biological or chemical event causes a change in the impedance (both resistance and reactance) at the liquid–electrode interface [38]. 

#### 3.1.1. Amperometric Detection 

**Figure 2:**
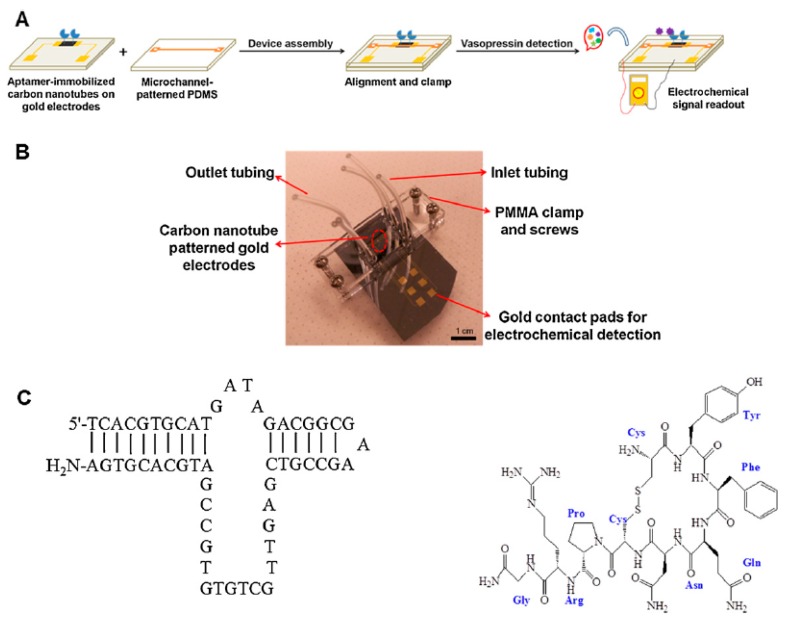
Aptamer-based amperometric microfluidic biosensor: (**A**) schematic of fabrication process of aptamer-based microfluidic biosensors; (**B**) a photo of the integrated microfluidic device; and (**C**) chemical structures of aptamer (left) and vasopressin (right). Reprinted from [40], Copyright 2013, with permission from Elsevier.

Amperometric detection is the first electrochemical technique adapted in microscale [30]. Amperometric biosensors are self-contained electrochemical devices that transduce the biological recognition events caused by the oxidation or reduction of an electroactive species into a current signal for the quantification of an analyte. The simple device architecture of this transduction approach makes it useful in low-cost portable devices for various applications including disease diagnosis and environmental monitoring [39]. He et al. used the amperometric detection technique to develop an aptamer-based label-free biosensor for the selective detection of vasopressin on a microfluidic platform to build a portable point-of-care (POC) diagnostic device [40]. As shown in Figure 2, carbon nanotubes (CNTs) were deposited between two lithographically patterned gold electrodes with a gap of 10 µm on a silicon wafer. The deposited CNTs were then modified with amine-terminated aptamer using carbodiimide and N-hydroxysuccinimide (EDC/NHS) surface chemistry. Afterwards, the aptamer modified device was integrated with a polydimethylsiloxane (PDMS)-based microfluidic channel for the continuous flow of vasopressin-containing fluid. The detection was performed by measuring the current before and after vasopressin binding. The specific binding between the aptamers and vasopressin depletes electron carriers on the CNT surface and reduce the local charge distribution resulting in a decrease in current magnitude. Using this technique, a limit of detection of 43 pM was achieved which is in the same order as the physiological level of vasopressin in the bloodstream. This miniaturized portable module has the potential to be a used as a point-of-care diagnostics tool for patients with excessive bleeding. 

#### 3.1.2. Voltammetric Detection

Voltammetric sensing is a technique where the electrical potential at the working electrode is scanned from one pre-set value to another and the electrochemical current is recorded as a function of the applied potential [41]. Unlike amperometric technique, this technique monitors the redox activity across a range of applied potentials manifesting well-defined current peaks [32]. Voltammetry is one of the oldest electrochemical techniques and has revolutionized the field of analytical chemistry. The main advantages of voltammetric sensors are their high sensitivity, reproducibility, easy implementation and low cost. Implementing voltammetric sensors was difficult in the past when computers were not readily available to precisely control potential scans. However, in the present, these techniques are increasingly becoming popular due to the advancement of portable computers and their ability to easily control and measure electrical signals [42].

**Figure 3:**
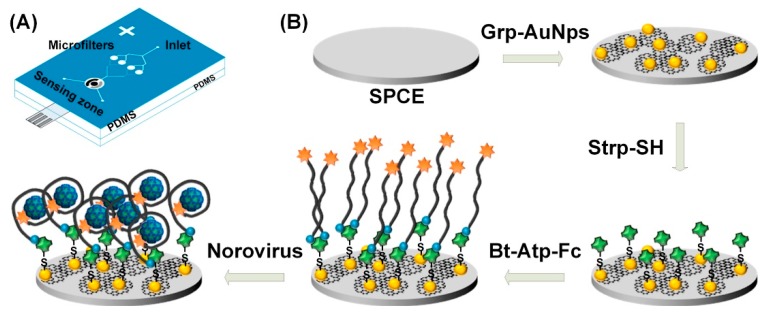
Microfluidic voltammetric aptamer-based detection of norovirus: Schematic of (**A**) the PDMS microfluidic chip; and (**B**) the processing steps to fabricate the biosensor. Grp-AuNPs: graphene-gold nanoparticles composite, Strp-SH: thiolated streptavidin, Bt-Atp-Fc: biotin and ferrocene tagged aptamer. Reprinted from [43], Copyright 2017, with permission from Elsevier.

Chand and Neethirajan from the Neethiarajan group have developed an aptamer-based voltammetric biosensor for the sensitive detection of norovirus on microfluidic platform using graphene-gold nanocomposites which offer the benefits of having enhanced electronic properties, high surface-area-to-volume ratio, and biocompatibility [43]. Built on a commercially available screen-printed carbon electrode (SPCE) for their cost-effectiveness and disposability, the PDMS-based microfluidic device has two inlets, a microbeads-based filtration zone, a sensing zone, and an outlet as shown in Figure 3A. Selective detection of norovirus was accomplished by immobilizing the ferrocene-tagged viral capsid-specific aptamer onto the surface-modified SPCE (Figure 3B). The interaction between the aptamers and norovirus results in a decrease in the voltammetric signal from ferrocene. Using differential pulse voltammetry (DPV), this microfluidic-integrated aptasensor was able to detect norovirus with a detection limit of as low as 100 pM and a detection range from 100 pM to 3.5 nM. 

Another voltammetric aptasensor with a linear detection range from 30 pg/mL to 10 μg/mL was recently developed by Sanghavi et al. for the detection of cortisol in biological media (serum and saliva) on microfluidics platform for point-of-care (POC) applications [44]. The working principle involves the displacement of triamcinolone (a drug used for skin treatment) that were initially bound to cortisol aptamers immobilized on gold nanoparticles. The displaced triamcinolone was electrochemically reduced at the graphene-coated glassy carbon electrode which produced a current that was proportional to the concentration of cortisol. This sensing approach only requires sample volumes below one microliter and has the ability to resist interferences from other glucocorticoids in the sample, and therefore holds promise for real-time sensing applications. Moreover, this method does not require a labeling, immobilization or the intermediate washing steps.

**Figure 4:**
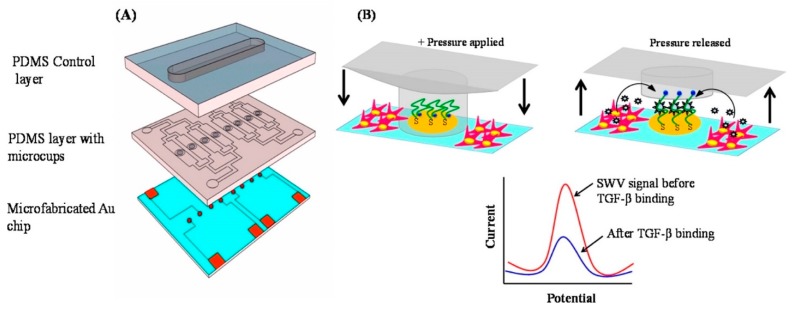
Aptamer-based monitoring of the TGF-β1 release from hepatic stellate cells on reconfigurable microfluidic platform: (**A**) the three layers of the microfluidic device. Bottom: glass slide with micropatterned Au electrodes. Middle: working PDMS layer with fluidic microchannels and microcups. Top: pressurizable control PDMS layer for controlling the microcups. (**B**) Diagram showing the actuation of the microcups. The plot gives a general description of the sensing mechanism. Reprinted with permission from [46]. Copyright 2016 American Chemical Society. https://pubs.acs.org/doi/10.1021/ac502383e (Further permissions related to this material should be directed to the ACS.)

Electrode fouling [45] often becomes a serious issue for electrochemical detection. To solve this problem, Matharu et al. have developed a reconfigurable microfluidics platform that uses aptasensors to electrochemically monitor the dynamics of the transforming growth factor TGF-β1 released by activated hepatic stellate cells over 20 hours [46]. This three-layer microfluidic device comprises a microfabricated gold electrode on glass slide, a microfluidic channel with microcups and a PDMS control layer (Figure 4). When the microcups were lowered by pneumatically actuating the control layer, the electrodes were protected during the protein adsorption and cell seeding steps. This reduced the non-specific adsorption of highly adhesive stellate cells, thus preventing electrode fouling. This work reported a 15-fold improvement in the sensitivity compared to traditional approached with unprotected electrodes. After seeding and activation of the stellate cells, square wave voltammetry (SWV) was applied to measure the electron transfer which resulted in a detection limit of 1 ng/mL.

#### 3.1.3. Impedimetric Detection

**Figure 5:**
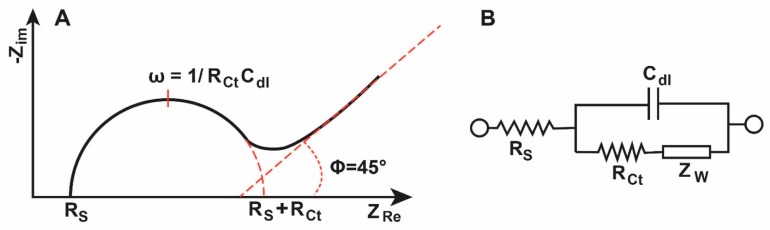
The Nyquist plot (**A**) and the corresponding Randles circuit (**B**).

An impedimetric transduction mechanism is a label-free mechanism that works on the basis of EIS. EIS can analyze both the resistive and capacitive properties of the electrode surface upon excitation and perturbation of the system at equilibrium with a small amplitude sinusoidal excitation signal [47]. The resulting spectrum (Figure 5A), known as the Nyquist plot, can be modeled by the Randles circuit as seen in Figure 5B. This Randles circuit is a valuable technique for the characterization, analysis and study of coatings, batteries, fuel cells and corrosion phenomena [47]. This circuit consists of a solution resistance (*R*_S_), a double-layer capacitance (*C*_dl_), a charge transfer resistance (*R*_ct_), and the Warburg impedance (*Z*_W_).

The resistor *R*_S_ is inserted as a series element to reflect that all the current must pass through the bulk solution. Moreover, the parallel elements are introduced since there are two possible current paths at the electrode–solution interface: one is the Faradaic charge transfer process and the other is the charge buildup due to the double-layer capacitance. Elements *C*_dl_ and *R*_ct_ are often used as the detection parameters in biosensing as they represent the dielectric and insulating features at the electrode–electrolyte interface, while *R*_S_ and *Z*_W_ depend on the bulk properties of the electrolyte and the diffusion of the redox probe, respectively [48]. 

**Figure 6:**
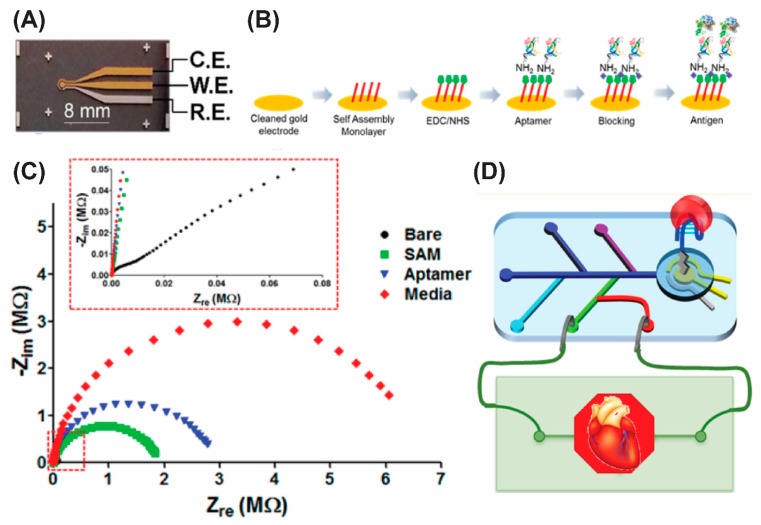
Aptamer-based impedimetric biosensor on microfluidics platform: (**A**) photograph of the microfabricated electrode set; (**B**) schematic diagram showing the immobilization and sensing steps of the biosensor; (**C**) electrochemical impedance spectroscopy (EIS) measurements of the biosensor at each modification and sensing steps; and (**D**) the cartoon showing the integrated heart-on-a-chip biosensor module. Reprinted with permission from [49]. Copyright 2016 American Chemical Society.

Shin et al. have integrated an aptamer-based impedimetric biosensor with microfluidic platform to develop an organ-on-a-chip biosensor for sensitive detection of a cardiac biomarker creatine kinase-muscle/brain (CK-MB) [49]. Here, the gold working electrode of a 3-electrode electrochemical cell (Figure 6A) was coated with a carboxy-terminated thiol followed by an immobilization of amine-linked aptamers via carbodiimide coupling (Figure 6B). The sensor was then exposed to culture media samples containing the CK-MB biomarker. The EIS measurements in Figure 6C show that detection of the biomarkers causes a significant increase in charge transfer resistance (*R*_ct_) resulting from the reduced charge transfer between the redox probe ([Fe (CN)_6_]^3−/4−^) and the electrode. Impedance signal (Δ*R*_ct_) for CK-MB was found to be linear in the clinically relevant concentrations from 10 pg/mL to 100 ng/mL in both buffer and culture media samples. The sensor was then integrated to a heart-on-a-chip cardiac bioreactor as seen in Figure 6D and doxorubicin-induced cardiac damage was evaluated by monitoring the changes in the creatine kinase concentration. 

**Figure 7:**
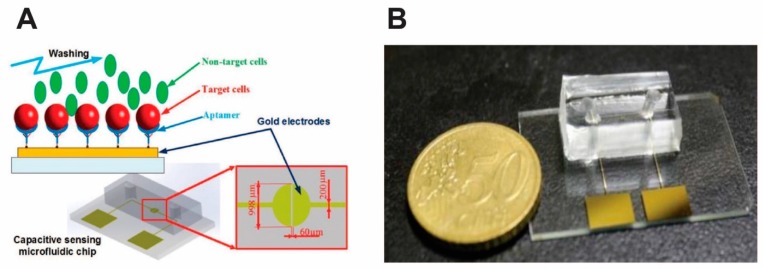
Schematic (**A**) and photograph (**B**) of the microfluidic biosensor chip. Reprinted with permission from [50].

Nguyen et al. have developed an impedimetric microfluidic-biosensor that uses the variation in capacitance of the Randles circuit as the sensing parameter for label-free selective detection of lung carcinoma cell line (A 549) [50]. As seen in Figure 7, a coplanar two-electrode configuration was used due to its simplicity of fabrication and ease of monitoring the change in material properties as the electric field is being applied between the electrodes. The gold electrodes were functionalized with aptamers and EIS was performed at frequencies ranging from 0.1 kHz to 1 MHz to monitor the occurrence of binding events. The capacitive response of the impedance was measured at various cell concentrations to evaluate the sensing performances of the device. A limit of detection of $1.5\times 10^{4}$ cells/mL was calculated. More recently, they have further upgraded their impedimetric biosensor by introducing gold nanoparticles into their system for enhanced sensitivity [51]. Here, the microfluidic biosensor uses 3-electrode configuration for the selective detection of the same A549 circulating tumor cells. Instead of complex EDC/NHS reaction, thiol-gold interaction was used to immobilize aptamers on the gold surface. Both of these biosensors developed by the Jen group can find applications in POC diagnostics for early-stage detection of lung cancer.

Lum et al. have also developed an impedimetric aptamer-based biosensor on microfluidic chip for specific detection of H5N1 avian influenza virus (AIV) on interdigitated gold electrode [52]. The aptasensor is capable of detecting AIV at concentrations as low as 0.0128 HAU (hemagglutinin units) in 30 minutes thanks to the use of interdigitated microelectrode that offers the advantages of low ohmic drop, increased signal-to-noise ratio, rapid reaction kinetics and hence low detection time. In contrast to commonly used square-shaped microfluidics chamber, an oval-shaped chamber was used here. An oval-shaped chamber offers the advantage of minimizing the residues, such as any non-specifically adsorbed aptamers or H5N1 virus that could potentially remain in the corners of the traditional square-shaped chambers. Thus, an oval-shaped chamber could contribute to the improvement of the detection sensitivity. This microfluidics-integrated aptasensor is portable, low-cost and has the potential to be used in rapid, in-field diagnostics. 

An interdigitated microelectrode was also employed by Lou et al. to develop a novel logic aptasensor (LAS) for intelligent detection of cancer cells utilizing a digital multimeter as a simple electrochemical read-out [53]. Integrated on a PDMS-based microfluidics platform, this biosensor is suitable for a simultaneous multi-analyte detection. Without any target cells, the two interdigitated gold-line microarrays behave as open circuits. However, in the presence of the target cells, the resistance across the electrodes reduces which can be measured by a handheld commercially available multimeter. The sensor is mass-scale producible and reusable enabling low-cost healthcare monitoring in POC testing and clinical applications. 

### 3.2. Microfluidic Aptasensors Based on Field-Effect Transistors (FETs) 

Field-effect transistors (FETs) have attracted much attention in the biosensing community as they offer many advantages such as miniaturization, low-cost, large-scale integration capability with the existing manufacturing process as well as label-free, rapid detection and highly sensitive detection of analytes [25]. A typical FET biosensor is composed of a semiconducting channel that connects the source and the drain electrodes. Any adsorption of biomolecules on the channel surface causes a change in the electric field that modulates the gate potential, resulting in a change in the drain current within the channel of the FET. Such change in the drain current can be used as a detection mechanism for the biosensors [54,55,56,57,58].

One-dimensional (1-D) nanomaterials such as silicon nanowires (SiNWs) [59], carbon nanotubes (CNTs) [60], and polymer nanowires [61] have long been explored as channel materials in FET-based biosensors due to their several advantages such as high switching characteristics (high current on-off ratio) and large specific surface areas. But their practical applications are limited by the major challenges that include device-to-device variations and relatively high cost in large-scale fabrication [58]. On the other hand, two-dimensional (2-D) nanomaterials such as graphene, MoS_2_, WS_2_, etc. are now becoming attractive alternatives to 1-D nanomaterials as a conduction channel for FET-based biosensors due to their planner structure, excellent electrical properties, high surface area-to-volume ratio, and easier large-scale manufacturability. Among several 2D materials, graphene has been the most widely used as a promising FET channel material due to its superior physical, chemical, electronic properties as well as its ability to be grown in large areas with high fidelity. For example, a graphene field-effect transistor (GFET) has been employed as a biosensor for detecting DNA [62], triphosphate [63], bacteria, and viruses [64,65] as well as protein biomarkers [25,66]. Integrating a GFET biosensor with a microfluidic device can produce synergistic effect from their respective advantages, further enhancing its sensing performances. 

**Figure 8:**
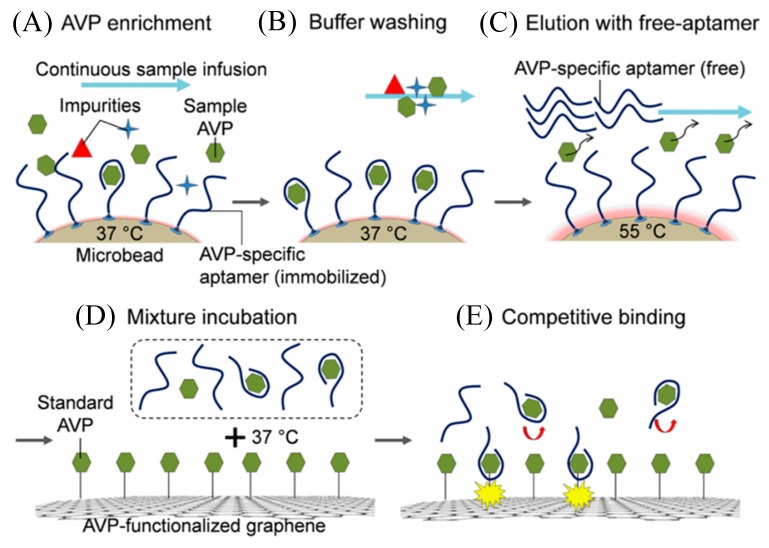
Schematics showing the working principle of the arginine vasopressin (AVP) detection. The sample AVP is enriched on microbead surfaces by aptamer binding (**A**) and then is washed with a buffer (**B**) followed by an increase in temperature to 55 °C that disrupts the aptamer-AVP complexes and release sample AVP into a free aptamer solution (**C**). The mixture of the free aptamers and the released sample AVP is incubated with graphene functionalized with standard AVP (**D**), that induces binding of free aptamer to the standard AVP on graphene via the competitive binding process (**E**) and thus changing the conductance of the graphene channel. Reprinted from [67], Copyright 2015, with permission from The 28th IEEE International Conference on Micro Electro Mechanical Systems (MEMS).

Here, we summarize the recently published work on GFET-based microfluidic biosensors. Yang et al. has built an integrated microfluidic platform that consists of (1) analyte enrichment using aptamers, (2) isocratic elution, and (3) a GFET-based nanosensor for the detection of arginine vasopressin (AVP) [67]. The working principle is illustrated in Figure 8 which shows the enrichment of the analyte (AVP) molecules through selective capturing by aptamers (Figure 8A), buffer washing (Figure 8B), isocratic elution (Figure 8C) process sequentially, resulting in a mixture (known as the elute) of the free aptamers and the released AVP originally captured from the sample (“sample AVP”). The elute is then incubated with graphene containing pre-functionalized with standard AVP. Through the competitive binding between standard and sample AVP, the change in the GFET current measurement indicates the sample AVP concentration (Figure 8E). This change in the channel conductance is used as an efficient interrogation probe for monitoring the presence of AVP concentration. The approach suggests the effectiveness of the GFET-based integrated microfluidics for achieving label-free detection of analytes. Wang et al. recently used a similar GFET-microfluidic platform for measurement of aptamer-protein binding kinetics [68].

**Figure 9:**
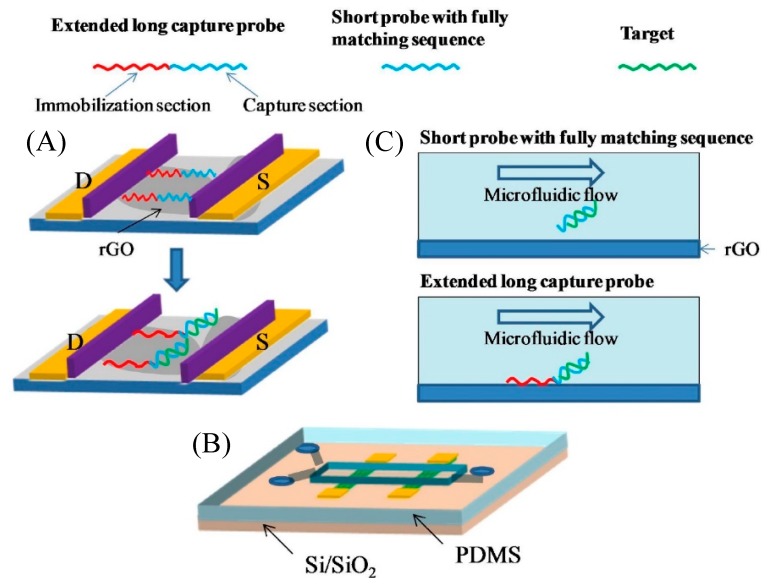
Mechanism of extended long-capture probe immobilization strategy to keep probe stability in the flow environment: (**A**) The immobilization section of extended long capture probe still keeps π−π stacking interaction on the reduced graphene oxide (rGO) surface after hybridization; (**B**) The PDMS microfluidic integrated rGO transistor chip; (**C**) In flowing environment, short capture probes with fully match sequence after hybridization with target are washed away from rGO surface. Extended long capture probes are still kept on rGO surface. Reprinted from [69], Copyright 2017, with permission from Elsevier.

Chan et al. have implemented a reduced graphene oxide (rGO) transistor integrated with microfluidics for the detection of H5N1 influenza virus gene using a flow-through strategy [69]. In a traditional approach, the short target capture probes that are immobilized by the π−π interactions are removed from the rGO surface by washing after hybridization. By contrast, the immobilization section of the extended long capture probe remains on the rGO surface leaving the π−π stacking interaction intact even after hybridization (Figure 9), resulting in a possible increase in the LOD. The sensing is monitored by monitoring the change in the drain current. On the other hand, there is negligible change in the drain current after binding with non-complementary target DNA which confirms the selectivity of the rGO FET biosensor. The rGO FET-based biosensor exhibited a detection limit of 5 pM with a typical detection time of 1 hour. 

### 3.3. Microfluidic Aptasensors Based on Optical Detection 

Optical detection is one of the most commonly used detection principles on lab-on-a-chip-based biosensors because it offers easy interfacing between microfluidic devices and the conventional optical-detection instruments commonly found in laboratories (such as inverted fluorescence microscopes, digital CCD cameras, simple light-emitting diodes (LEDs), laser diodes and photodiode set-ups and even smartphones) [34]. Some examples of an optical detection include measurements based on absorbance, fluorescence, chemiluminescence, colorimetry, interferometry, and surface plasmon resonance.

#### 3.3.1. Fluorescence Detection

Among different optical detection mechanisms, fluorescence-based detection is by far the most widely used sensing technique encountered in microfluidic applications thanks to its ease of implementation. One of the common fluorescence-based techniques works on the basis of the Förster resonance energy transfer (FRET). FRET is based on the coupling between a fluorophore (visible light-emitting molecule) and a quenching molecule that absorbs visible light and emits optical energy at invisible wavelengths. Although these fluorescence biosensors offer multiple advantages such as very low detection limits, high selectivity, and availability of a wide array of fluorescence labels for tagging biomolecules, quantitative analysis can be difficult due to the interference caused by the auto-fluorescence from the microfluidic chip material in case of polymeric devices [34].

One of the most widely used nanomaterials in aptamer-based fluorescence biosensors is graphene oxide (GO). GO is a graphene nanosheet containing large amounts of oxygen atoms in the form of epoxy, hydroxyl, and carboxyl groups [70]. The main reason for its widespread use, especially in the biosensors area, is its high optical quenching ability, excellent dispersibility in aqueous media, biocompatibility and easy surface functionalization capability [71]. GO interacts with single-stranded DNA through π−π stacking with the nucleotide bases and effectively quenches the fluorescence of quantum dots (QDs) [72,73]. The quenching mechanism is based on the principle of photoinduced electron transfer or energy transfer. 

**Figure 10:**
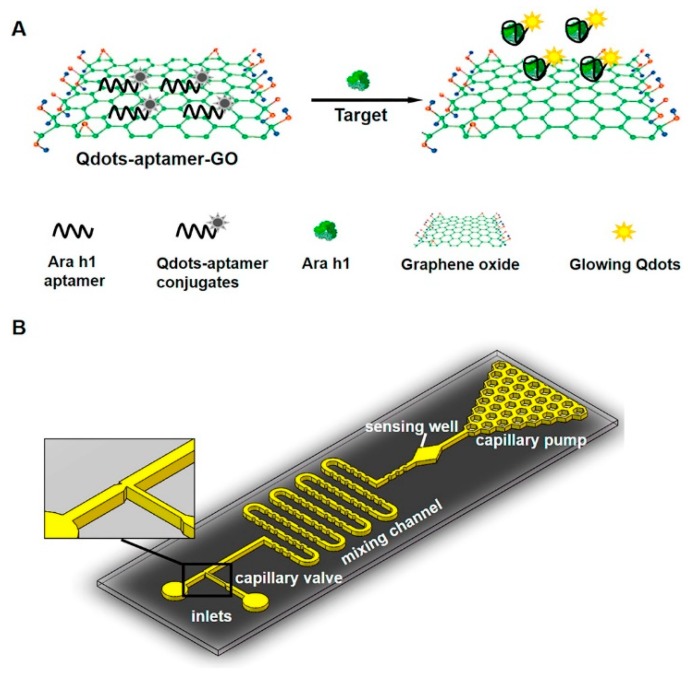
Microfluidic fluorescence biosensor: Schematic diagram of the (**A**) sensing mechanism of the quantum dots (QDs)-aptamer-graphene oxide (GO) quenching system; and (**B**) microfluidic chip which has two inlets for loading the QDs-aptamer-GO probe mixture and the Ara h 1 sample, respectively. Reprinted from [74], Copyright 2016, with permission from Elsevier.

Neethirajan group had extensively exploited the exceptional quenching capability of GO to develop several aptamer-based biosensors on lab-on-a-chip platform [74,75,76]. One example is the aptamer-based biosensor on PDMS/glass microfluidic chip for simple, rapid, and sensitive food allergen (Ara h1) detection developed by Weng and Neethirajan [74]. This device utilized quantum dots-aptamer–GO complexes (QDs-aptamer-GO) as probes to undergo conformational changes upon interaction with the food allergens. As can be seen in Figure 10, in the absence of the target analyte, the fluorescence of the QDs is quenched via the FRET process between the (QDs)-aptamer probes and GO due to their self-assembly through specific π−π stacking interaction, resulting in no fluorescence signal. Upon binding the target analyte, QDs-aptamer probes are released from the GO leading to the recovery of fluorescence of QDs. The same principle was used by Weng and Neethirajan [75] to develop another aptamer-based biosensor for rapid, and sensitive detection of food allergens and food toxins. In this device, a PDMS/paper microfluidics platform was used instead of the PDMS/glass microfluidics. 

First proposed by Whitesides and co-workers [77], paper-based microfluidic devices, commonly referred to as microfluidic paper-based analytical devices (µPADs), has rapidly emerged as an alternative approach to the traditional microfluidic devices based on glass, silicon, PDMS or other polymeric materials. Paper is a ubiquitous material and is comprised of biocompatible porous cellulose fiber web with large surface area. Its porous nature fulfils the primary task of fluid transport through the predefined channel. Moreover, µPADs are powerless due to the capillary action of the hydrophobic channel [76]. The success of µPADs can be attributed to three main factors: low cost, simple operation, and the ability to manipulate fluid flow without any external pumps [78]. Therefore, Weng and Neethirajan [76] developed an aptamer biosensor on a paper-based microfluidic platform for sensitive detection of norovirus. Instead of using QDs, 6-carboxyfluorescein (6-FAM) was used as the aptamer label, and multi-walled carbon nanotubes (MWCNTs) and graphene oxide were used for the quencher in their devices. In the presence of the target norovirus, the labeled aptamers were released from the MWCNT surface resulting in the recovery of a fluorescence signal, which was subsequently measured by the multi-mode reader. 

Liang et al. have developed another fluorescent and multiplexed aptasensor for monitoring cancer cells in µPADs that can be observed by a naked eye [79]. The device utilizes quantum dots-coated mesoporous silica nanoparticles-labeled aptamers that are attached to the surface of GO. The fluorescence that was initially quenched via FRET was subsequently recovered upon addition of the target cells. The unique design allows for alteration of other target cancer cells by simply utilizing the change of the aptamer and different colored QDs. With this technology, simultaneous detection of three different cancer cells was achieved with LODs of 62, 70, and 65 cells/mL for MCF-7, HL-60, and K562 cells, respectively. Furthermore, the work contributed to clinical sample analysis with satisfactory results. Similar FRET-based detection mechanisms were also adopted by Ueno et al. [80] to develop a linear array GO aptasensor for multiplexed detection of three protein biomarkers: thrombin, prostate specific antigen (PSA) and hemagglutinin (HA). 

**Figure 11:**
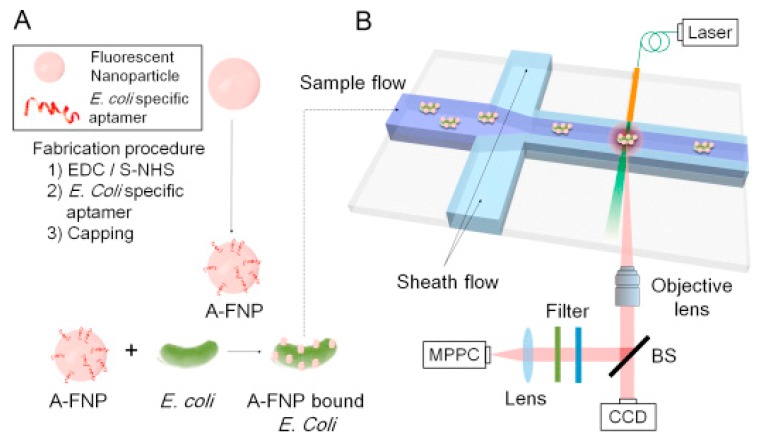
Schematics of the aptamer-based optofluidic detection system: (**A**) preparation of aptamer conjugated fluorescence nanoparticles (A-FNPs); and (**B**) detection of A-FNP-bound *E. Coli* by the microchannel and optical particle counter. Reprinted from [81], Copyright 2015, with permission from Elsevier.

Chung et al. have developed an optofluidic system that uses target specific aptamer-conjugated fluorescence nanoparticle (A-FNP) for fast and continuous detection and monitoring of airborne microorganism *E. Coli* [81]. In this real-time and non-destructive single cell detection system, fluorescence nanoparticles are attached to the surfaces of bacterial cells causing them to be optically excited with the wavelength of 540 nm. The developed system consists of a PDMS microchannel having 3 inlets (1 sample flow and 2 sheath flows), 1 outlet, and an optical detector (Figure 11). The sensing was accomplished by exciting the A-FNP-labeled *E. Coli* cells in the detection zone with a fiber-coupled laser and the emitted fluorescence was quantitatively measured with a photon counter. This optofluidic system resulted in a detection throughput of up to ~100 particles per second with 85% accuracy.

Aptamer-based sandwich immunoassay techniques were developed by several research groups to design fluorescence-based aptamer biosensors for rapid sensing of analytes on microfluidics platform. In this assay, two aptamers are required: one is the capture aptamer and the other one is the reporter aptamer. The capture aptamer is immobilized onto a substrate and interacts with the target analyte. Subsequently, the reporter aptamer which is tagged with a certain label (i.e. fluorophore) binds with the captured analyte, resulting in a sandwich structure. For example, Tseng et al. used sandwiched aptamer-based assay for automatic fluorescence detection of H1N1 influenza virus on microfluidic platform [82]. This microfluidic system was designed to automatically carry out the entire detection process in less than 30 minutes which is significantly faster than the conventional viral culture method. A limit of detection of 0.032 hemagglutination unit (HAU) was achieved thanks to the high affinity and high specificity of the H1N1-specific aptamers. Furthermore, this two-aptamer microfluidic system had approximately 3 orders of magnitude higher in sensitivity than the conventional serological diagnosis. Li et al. have also developed an integrated microfluidic system for rapid and automatic measurements of glycated hemoglobin using the sandwich-based approach [83]. The two parallel assays were developed for quantifying glycated hemoglobin and total hemoglobin. This assay technique took 86% shorter analysis time than the conventional HPLC-based approach as well as 75% less reagent consumption.

**Figure 12:**
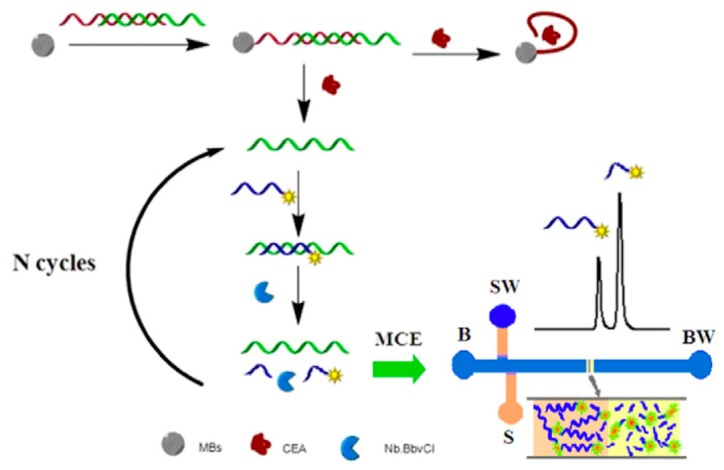
Schematic illustration of detection principle with the proposed aptamer-based microfluidic capillary electrophoresis (MCE) assay for amplification detection of carcinoembryonic antigen (CEA). Abbreviations: S, sample reservoir; SW, sample waste reservoir; B, buffer reservoir; BW, buffer waste reservoir; MBs, magnetic beads. Reprinted from [85], Copyright 2015, with permission from Elsevier.

Microfluidic capillary electrophoresis (MCE), a miniaturized platform of capillary electrophoresis (CE), can provide a simple and convenient solution for biochemical analysis compared to other methods such as high-performance liquid chromatography (HPLC), gas chromatography and CE [84]. Several research groups have exploited this MCE platform in conjunction with fluorescence detection system to develop aptamer based microfluidic biosensor [85,86,87]. For example, Pan et al. [85] used this platform for rapid detection of tumor marker carcinoembryonic antigen (CEA). A laser-induced fluorescence (LIF) detection system was used to detect CEA in serum samples from colorectal cancer (CRC) patients and healthy subjects. As seen in Figure 12, a double-stranded DNA (dsDNA), which was formed by an anti-CEA aptamer and its complementary DNA (cNDA), was conjugated with a magnetic bead (MB). In the presence of the target CEA molecule in the sample, the cDNA was released and hybridized with a fluorescein amidite (FAM)-labeled DNA forming a DNA duplex. This DNA duplex triggers the selective cleavage of the FAM-labeled DNA probe by nicking endonuclease (Nb.BbvCI), resulting in a continuous release of the cDNA. The released cDNA was repeatedly hybridized with another FAM-labeled DNA that led to the generation of large numbers of FAM-labeled DNA segments, resulting in an amplification of the fluorescence signal. Afterwards, the MCE laser-induced fluorescence (MCE-LIF) detection system was able to separate and detect the FAM-labeled DNA segments. The use of magnetic beads is to assist in the target-induced strand cycle that would lead to an increase in the optical sensitivity. Using this MCE platform, a limit of detection of 68pg/mL was obtained with a linear range of 130pg/mL–8 ng/mL, making it a promising technology for use in clinical diagnosis, prognosis, and monitoring of cancer progression in CRC.

The MCE platform was also used by other research groups such as in Wang et al. [87] who utilized it to develop an aptamer-based fluorescence detection of multiple antibiotic residues in the field of food safety screening. The MCE platform was combined with catalyzed hairpin assembly (CHA) as CHA can act as a signal amplifier without the need for a label, which may decrease the performance of the aptamers or negatively impact the self-assembly of hairpins. On the other hand, He et al. combined MCE with stir bar-assisted sorptive extraction and rolling circle amplification (RCA) to implement a ratiometric fluorescence biosensor for highly sensitive detection of antibiotics in food [88]. Unlike conventional aptamer-based biosensors, this ratiometric sensing can be employed to detect samples in a complex matrix such as foods and soils and can avoid potential problems of matrix interference or sample-to-sample variations. 

#### 3.3.2. Colorimetric Detection

**Figure 13:**
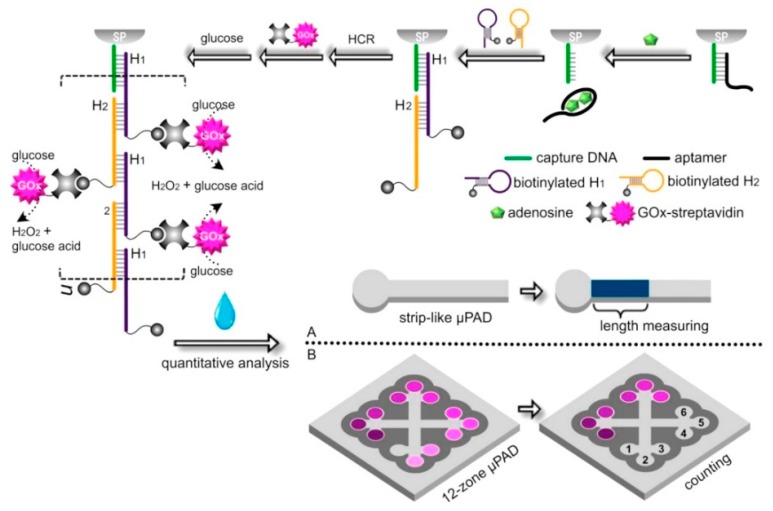
Schematic diagram illustrating the naked-eye quantitative aptamer-based assay for the detection of adenosine as a model analyte based on (**A**) the length measurement of the colored region in a strip-like microfluidic paper-based analytical device (μPAD) or (**B**) the counting of the colorless microzones in a wax-pattered 12-zone μPAD. Reprinted from [90], Copyright 2016, with permission from Elsevier.

One of the main advantages of colorimetric detection is that the color detection can be easily achieved with various commercial devices such as smart phones and flatbed scanners [89]. It is often considered a simple and low-cost method to measure quantitative output signal. However, its lack of sensitivity limits its usage in applications requiring high accuracy such as the detection of rare cancer cells in blood sample [53]. Recently, Zhang et al. created an aptamer-based colorimetric biosensor for achieving naked eye quantitative assay on paper, which relies on measuring the length of the colored region in a strip-like µPAD or counting the number of colorless detection microzones in a multi-zone µPAD [90]. Figure 13 shows the schematic illustration of the naked-eye aptamer-based paper microfluidic assay for the detection of adenosine based on the measurement of a colored region as well as counting of the colorless microzones in a wax-patterned 12-zone μPAD. For test results, a naked-eye quantitative readout is performed either by simply measuring the length of the colored region of the strip-like µPAD or by counting the number of colorless detection microzones. These equipment-free and quantitative methods hold great potential in the commercialization of aptamer-based POC devices that are low-cost, portable and user-friendly. 

Another μPAD-based colorimetric aptasensor was developed by Wei et al. [91] for the simultaneous detection of cocaine, adenosine, and lead ion. A target-responsive hydrogel was used as flow regulator and controller. In the absence of a target, the hydrogel formed in the channel blocks the flow causing a “signal off” readout. By contrast, in the presence of the target, the hydrogel-free fluidic channel allows the colorimetric indicator to reach the observation spot producing a “signal on” readout. The entire assay can be completed within 6 minutes and the observation can be made by the naked eye without any auxiliary equipment. The reported detection mechanism offers a simple, cost-effective, rapid, and user-friendly POC testing device to be used in various applications. 

**Figure 14:**
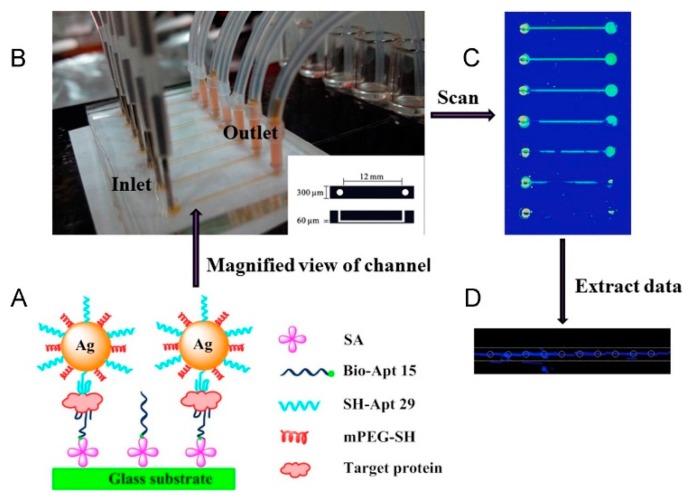
Colorimetric detection using silver nanoparticles aptasensor: (**A**) schematic illustration of capturing target proteins and colorimetric detection based on aptamer-AgNPs in the microfluidic chip; (**B**) photograph of the microfluidic device consisting of 7 channels for thrombin capture and colorimetric detection; (**C**) scanned picture of microfluidic chip after reaction; and (**D**) gray scale values were acquired on each channel. Reprinted from [89], Copyright 2016, with permission from Elsevier.

Nanoparticles are often employed in colorimetric detection because of their inherent optical or catalytic properties. Several works have been published on the colorimetric detection using nanoparticles [89,92,93,94]. Zhao et al. used silver nanoparticles (AgNPs) to develop a naked-eye colorimetric aptasensor on glass/PDMS microfluidic platform for the simple detection of protein biomarker thrombin [89]. Surface-functionalized microfluidic channels were used as the capture platform (Figure 14). The introduction of thrombin into the channel forms a sandwich-type complex involving immobilized AgNPs. Increasing concentrations of thrombin cause an increase in the amount of aptamer-AgNPs on the complex resulting in colorimetric changes that can be observed either by a naked eye or a flatbed scanner. Thrombin concentrations of as low as 20 pM have been detected using this aptasensor device without any signal amplification.

Colorimetric assays in conjunction with nucleic acid amplification have been used extensively which offer enhanced detection capabilities. As an example, Lin’s group developed a portable glass/PDMS microchip to realize highly sensitive detection of thrombin protein by integrating it with rolling circle amplification (RCA) [95]. Thrombin was first isolated using aptamers and the secondary thrombin aptamers with a G-quadruplex template was used to amplify the signal using RCA. The reported detection limit was 0.083 pg/mL measured from thrombin in plasma and serum. 

**Figure 15:**
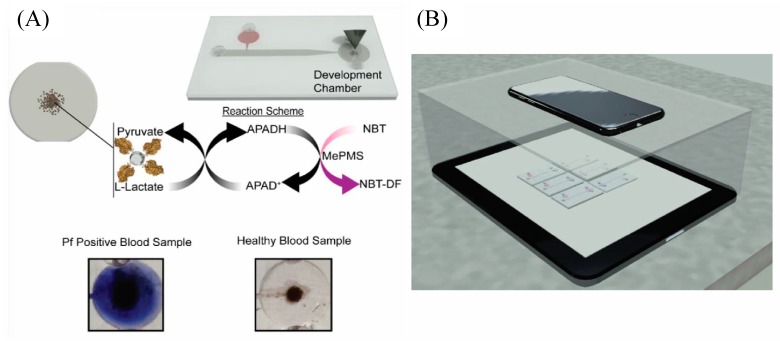
Microfluidic aptamer-tethered enzyme capture (APTEC) colorimetric biosensor: (**A**) the reaction scheme of the reagents and redox reaction that results in the generation of an insoluble purple diformazan dye. There was a color difference between positive and negative samples; and (**B**) the smartphone camera was used for capturing images in a telemedicine application. Reprinted from [96], Copyright 2018, with permission from Elsevier.

A colorimetric biosensor for telemedicine application has been developed by Fraser et al. [96] who have integrated an aptamer-tethered enzyme capture (APTEC) technique on a microfluidic device for magnet-guided capture, wash, and detection of the biomarker. Three separate microfluidic chambers were designed to implement the magnet-guided colorimetric detection of the PfLDH protein. In the first chamber (incubation chamber), aptamer-coated micromagnetic beads (μMBs) were bound specifically to their target by incubation in a lysed sample of human blood. Next, a washing step was conducted in the second chamber to remove any unbound molecules and other contaminants. This was followed by the transfer of the aptamer-μMB-target complex to the third chamber containing the development reagent that generated a strong colorimetric signal in the presence of PfLDH. (Figure 15A). The signal analysis was conducted by placing the device on top of a tablet device (Figure 15B) displaying a homogeneous background in a concealed box. Images were captured using a smartphone camera and coupled with metadata containing time, date, and GPS coordinates for the telemedicine application. At the receiver side, the images were analyzed with ImageJ software.

#### 3.3.3. Chemiluminescence and Electrochemiluminescence-Based Detection

**Figure 16:**
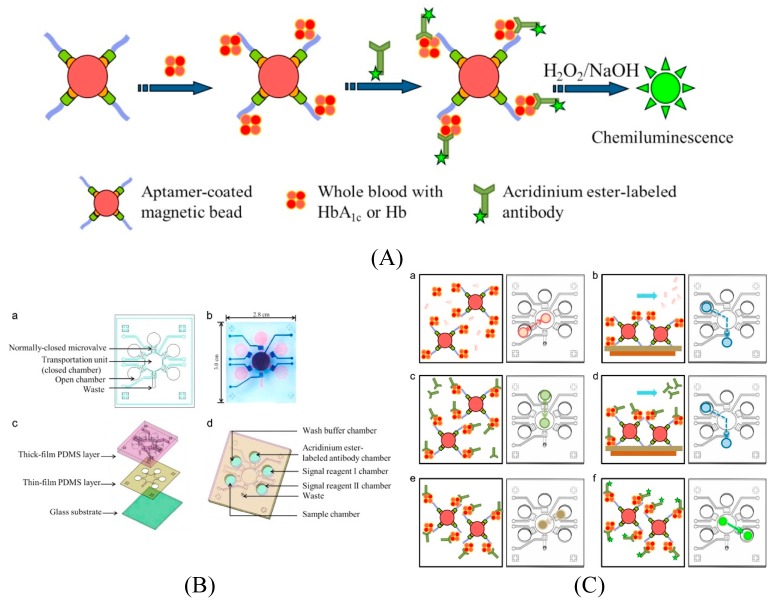
Chemiluminescent aptamer-based microfluidic biosensor: schematic illustration of the (**A**) working principle; (**B**) (**a**–**d**) microfluidic chip composed of micro-components; and (**C**) experimental procedure performed on the integrated microfluidic chip. (**a**) magnetic beads pre-coated with Hb or HbA1c aptamers were transported to the transportation unit (the close chamber) and incubated with blood samples; (**b**) target-aptamer-bead complexes were collected by applying an external magnetic field while washing the supernatant and non-binding substances with phosphate-buffered saline (PBS); (**c**) the acridinium ester-labeled Hb or HbA1c specific antibodies were transported to the transported to the transported unit and reacted with Hb or HbA1c; (**d**) the sandwich-like structures with target-aptamer-bead complexes were collected while washing the supernatant and non-binding substances with PBS buffer; (**e**) H_2_O_2_ was transported to the transportation unit to re-suspend the magnetic beads; and (**f**) the target-aptamer-bead complex with the acridinium was transported to the NaOH chamber and the chemiluminescent signals were detected with a luminometer. Reprinted from [99], Copyright 2015, with permission from Elsevier.

Luminescence is a phenomenon where certain molecules emit light not resulting from heat [97]. Luminescence can be of different types depending on the source of energy or the trigger for luminescence, some examples being chemiluminescence (CL) and electrochemiluminescence (ECL). Due to their considerable advantages including sensitivity, selectivity, and large linear quantitative range, luminescence-based detection is becoming increasingly popular for biosensing applications [98]. For example, as no excitation instrumentation is required, the background noises in CL signals are significantly reduced increasing the sensitivity compared to assay performed on bench-top equipment [99]. Chang et al. have developed an aptamer-based biosensor on a microfluidic platform using CL detection scheme for selective detection of blood glycated hemoglobin (HbA1c) [99]. Figure 16 illustrates the sensing principle as well as the analysis procedure for the integrated microfluidic chip. First, Hb- or HbA1c-specific aptamers immobilized on magnetic beads (MBs) were transported to the chamber where they were incubated with blood samples (a). Following a washing step to remove the unbound fractions by PBS buffer and aided by an external magnet (b), the target-aptamer-MB complexes were incubated with acridinium ester-labeled Hb or HbA1c specific antibodies to form aptamer-antibody sandwich-like structures (c). In the following step, another washing step was performed to remove the unbound antibodies magnetically (d). Finally, H_2_O_2_ was injected into the transportation unit to re-suspend the sandwich-like complex magnetic beads (e) which was then followed by the transporting of target-aptamer-bead complex with acridinium to the NaOH chamber where CL signals were monitored with a luminometer (f). Giuffrida et al. have reported a chemiluminescent sensor that employs digital microfluidics for aptamer-based detection of human lysozyme [100]. With this droplet microfluidics-based technique that uses gold nanoparticles as an enhancement agent for the CL signal, a detection limit of 44.6 fM was achieved using a sample volume as low as 1 µL and a detection time in the range of 10 minutes. 

ECL is a powerful analytical technique which is gaining increased attention for the development of analytical methods using µPADs [79]. One example is the ECL-based µPAD developed by Ma et al. [101]. This work uses the specific recognition by aptamers and the amplification strategy of a hybridization chain reaction (HCR) with the ECL probe, Ru(phen)_3_^2+^ for detecting the analyte peptides. 

Recently, Lin et al. have developed a “turn-on” aptasensor on microfluidics platform for sensitive detection of vascular endothelial growth factor VEGF165 that employs iridium (III) complex as a novel G-quadruplex-selective luminescent probe [102]. In this work, a hairpin-structured functional nucleic acid (FNA) was constructed which consisted of a VEGF165 aptamer and a G-quadruplex sequence as well as its complementary sequence. Upon binding to VEGF165, the functional nucleic acid rearranges its structure to form a G-quadruplex structure which is able to bind with the luminescent iridium (III) complex to report the analyte. The hairpin structure is unable to bind to iridium (III) complex when VEGF is absent. This luminescence-based aptasensor resulted in a specific detection of VEGF165 with low background, a linear range of 0.52–78.0nM, and a LOD of 0.17pM.

#### 3.3.4. Optical Interferometric Detection 

Interferometry offers the possibility of label-free, highly sensitive detection of biomolecules. In general, interferometric biosensing techniques work by measuring small changes that occur in an optical beam (sensing beam) along its path of propagation. When any binding event takes place, it causes a change in the refractive index along the optical path, resulting in a phase shift in the sensing beam with respect to the reference beam. By imaging the interference pattern resulting from the projection of these two beams, one can directly monitor any binding event that occurs in the biosensor [28,103]. 

**Figure 17:**
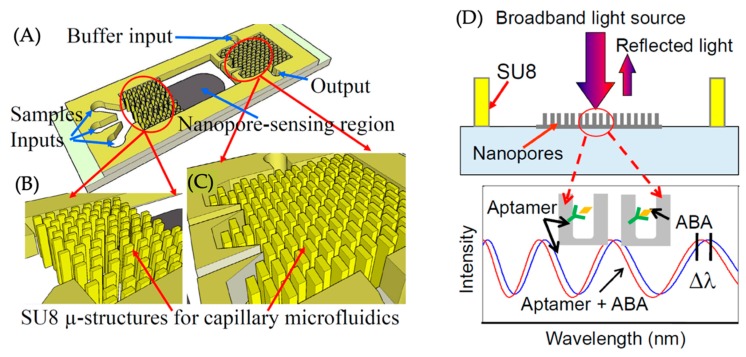
Optical interferometric detection-based biosensor: (**A**) schematic of the sensor chip; (**B**,**C**) enlarged view of the SU8 microstructures for microfluidics capillary interface; (**D**) the optical transducing signal from the nanopore-sensing region. Reprinted from [104], Copyright 2017, with permission from the 30th IEEE International Conference on Micro Electro Mechanical Systems (MEMS).

Song et al. have reported an optical interferometric aptamer-based biosensor for sensitive and specific detection of plant hormone abscisic acid (ABA) on a microfluidic device [104]. As can be seen in Figure 17, the microfluidic chip consists of a nanopore-based sensing region and SU-8 microstructures as capillary microfluidics on both sides of the sensing region consisting of periodically distributed nanopores. These nanopores are used to generate the optical interference fringes that work as the transducing signal for the biosensor. Upon delivering the samples to the chip, the capillary force generated from the SU-8 nanostructures automatically transport the samples to the nanopore sensing region. Upon specific binding between the ABA and the aptamer, a phase shift in the transducing signal occurs. Not only did this sensing strategy offered exceptional sensitivity in ABA detection, it also enabled the automation of the total sensing process without any external pumps.

#### 3.3.5. Surface Plasmon Resonance (SPR)-Based Detection 

**Figure 18:**
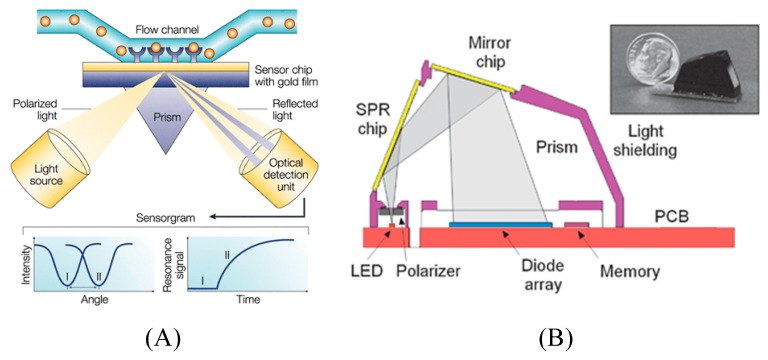
Principle of surface plasmon resonance (SPR) biosensor: (**A**) schematic of a SPR-based detection method. Reprinted from [107], Copyright 2002, with permission from Springer Nature; and (**B**) cross-sectional view of the Sensata Spreeta SPR sensor. Inset shows the photograph of the actual device. Reprinted from [108], Copyright 2003, with permission from Elsevier.

Surface plasmon resonance (SPR)-based sensing has received increased popularity not only in gas sensing, but also in many other applications including in medical diagnostics, food safety, and biology [105] due to its advantages that enable label-free detection, real-time detection, high sensitivity as well as its ease of preparation [106]. SPR is an optical technique that relies on the change in the refractive index of a metal surface on which the target molecules are immobilized. Upon incidence of light on a thin metal film at a specific angle directed by a prism, it excites a special electromagnetic wave known as surface plasmon at the surface of the metal. Because this incident angle is highly sensitive to the dielectric environment on the opposite side of the metal, any binding event on the metal surface causes the angle to shift (from I to II in the lower left-hand diagram of Figure 18A). This shift can be measured non-invasively in real-time [28,107]. 

Several laboratory scale SPR detection systems for immunosensing and DNA sensing has already been developed, most notably the Biocore from GE Healthcare. However, recently, efforts have been focused on reducing the size and complexity of the SPR by integrating the sensing component with microfluidics for more accurate diagnostic and automated measurements. One such example is the commercially available Sensata Spreeta SPR [108] sensor (Figure 18B) developed by Waswa et al. [109] for immunological detection of *E. coli* O157:H7 in milk, apple juice and ground beef extract. Using the same instrument, a similar assay was designed by Wei et al. [110] to detect Campylobacter jejune in poultry milk. Digital microfluidics-based SPR has also been implemented to further reduce the sample volume which is particularly important for cases where the analyte sample volume is scarce and precious [111,112,113].

So far, significant effort has been directed towards developing metal-based biosensors at visible and near-infrared optical spectra [114,115,116,117,118]. However, this was challenged by the intrinsic drawbacks, such as the high intrinsic hydrophobicity, the high surface inertness, and the large electronic density of states, presented by the traditional noble metals. Therefore, researchers have been in search for new materials that can overcome those limitations. Graphene, an emerging two-dimensional material, offers many unique opportunities to address these challenges. Compared to metals, graphene supports the propagation of surface plasmons at infrared spectrum with relatively low loss and high confinement. In addition, graphene enables efficient adsorption of biomolecules on its surface due to its high surface-to-volume ratio and the π–π stacking interactions with the biomolecules. Furthermore, an external gate voltage can be used to modulate the frequency of the surface plasmonics in the infrared spectrum easily [106]. Wei et al. proposed a cavity-enhanced infrared sensor based on graphene surface plasmonics [119], wherein a continuous and non-patterned graphene that excites the surface plasmon on its surface was used as the sensing medium.

In order to make the SPR-based biosensors compatible with lab-on-a-chip applications, microfluidics is often integrated with the SPR. In particular, membrane based microfluidic devices have widely been used in the field of diagnostics due to their simple and flexible design, easy construction and cost-effectiveness [120]. As an example, Chuang et al. have introduced a membrane-based microfluidic device integrated with a SPR sensor for quantitative detection of interferon gamma (IFN-γ) [120]. This disposable low-cost microfluidic aptasensor was designed in such a way that each step of washing, detection, and signal amplification had a separate fluid control mechanism. The sensing procedure was initiated by depositing the washing solution onto the transport membrane for wetting the rayon membrane. The sample solution was then flown through the sensing area and was contained in the transport membrane. Afterward, a controlled amount of streptavidin was introduced to amplify the SPR signal. Any unbound sample and streptavidin were removed through a washing step. This sensor was able to perform IFN-γ detection with a limit of detection of 10 pM within 30 minutes.

Although SPR is a well-established technique with excellent sensitivity, some minor disadvantages include the requirement of a thin layer of metal (typically gold), thus raising manufacturing cost, and its strong dependence on temperature variation [28].

### 3.4. Microfluidic Aptasensor Based on Mass-Based Detection 

Gravimetric or mass-based biosensors work on the basic principle of measuring the change in the mass at the sensing surface caused by the binding of the analyte to the receptors. Most gravimetric biosensors rely on the use of piezoelectric quartz crystals which can be used in two main formats: as resonators or as surface acoustic waves [121]. The QCM biosensors have been very popular in the area of rapid detection of pathogens [122] and toxins [123] because of their multifarious advantages such as ease of use, shorter analysis time, low-cost, as well as the possibility of label-free and real-time detection. On the other hand, surface acoustic wave (SAW)-based biosensors can detect acoustic waves generated by the interdigital transducers (IDTs) which are periodic metallic bars deposited on a piezoelectric material. Upon recognition of an analyte by the immobilized receptors, the velocity of the SAW changes that produces signal by the driving electronics.

#### Surface Acoustic Wave (SAW)-Based Biosensor 

The SAW, which was first described by Lord Rayleigh, refers to the propagation of an acoustic wave along the surface of a piezoelectric material [124]. However, SAW devices could not play an important role in practical applications until White and Voltmer proposed a technique to generate a SAW using interdigital transducers (IDTs) [125] fabricated on the surface of a piezoelectric material. Application of a sinusoidal wave whose period matches the grating period of the IDTs creates a vibration beneath the IDTs generating an acoustic wave that propagates along the surface perpendicular to the direction of the digits in the IDTs. As the wave velocity in the piezoelectric material is 10^−5^ times smaller than the electromagnetic wave, the wavelength of the SAW is shorter than the electromagnetic waves by 5 orders of magnitude making it a compact device [124]. 

**Figure 19:**
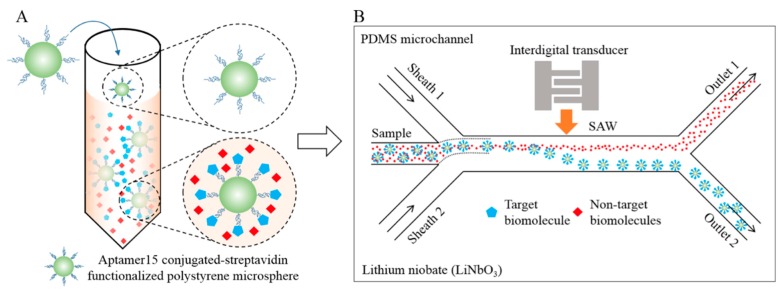
Schematic illustration of the acoustofluidic separation of target protein from a sample mixture. (**A**) A biotin-labeled aptamer conjugated to streptavidin-functionalized microparticles (green) specifically captured the target biomolecules (blue) from a complex mixture. (**B**) Microfluidic separation of target biomolecules from the non-target ones (red) using surface acoustic waves (SAWs) originating from the interdigital transducers (IDTs). Reprinted with permission from [127]. Copyright 2017 American Chemical Society.

SAW biosensors have been applied in clinical diagnosis due to their inherent advantages of high sensitivity, low cost, low power requirement and real-time monitoring capability. The integration of SAW technologies with microfluidics has created a new field called acoustofluidics that offers continuous, rapid and real-time monitoring of biomolecules as well as separation of microparticles based on size [126,127]. As an example, Ahmad et al. [127] have proposed a microfluidic device for size-based acoustofluidic separation of target proteins conjugated to microparticles. This platform contained a PDMS microchannel with an interdigitated transducer which was patterned on top of the piezoelectric lithium niobite (LiNbO_3_) substrate as a source of high-frequency SAWs. The target-specific aptamer conjugated to streptavidin-functionalized polystyrene microparticles was incubated in the sample mixture and was allowed to form the microparticle-aptamer-target complexes. (Figure 19A). Upon application of high-frequency SAWs, the acoustic radiation force caused the microparticle-aptamer-target complexes to deflect laterally and separate from the mixture to be collected at outlet 2 (Figure 19B).

Love-wave sensors, which are a special type of SAW sensors, offer increased surface sensitivity by reducing energy dissipation of the acoustic wave into the fluid. They are effective in applications requiring very high sensitivity for detection of mass loadings in liquids [126]. Zhang et al. proposed a microfluidic aptamer-based love-wave biosensor for real-time monitoring of prostate specific antigen (PSA) [126]. This SAW-based sensor consists of LiTaO_3_ substrate with two sets of IDTs patterned on SiO_2_ wave guiding layer as well as a gold film as an aptamer immobilization layer. Subsequently, a PDMS-based microfluidic channel was fabricated to allow liquid flow between the IDTs. Two SAW resonators were fabricated: one acting as a reference and the other as a sensing component. Under this platform, a detection limit of 10 mg/mL was achieved. 

## 4. Conclusion and Outlook 

This review summarizes the current state-of-the-art LOC systems that utilize aptamer-based biosensors. Table 1 summarizes them in terms of the device features, target analytes, matrix samples, as well as their microfluidic channel materials. The integration of aptasensors to various forms of microfluidics and paper-based analytical devices have further increased the versatility of aptamers. Although the technology seems promising for potential clinical use, there are still challenges remaining that must be addressed for it to revolutionize the healthcare and diagnostics sector. Designing and selecting a high-affinity aptamer is not a trivial task. Indeed, there are still many biomarkers of critical importance that do not have corresponding aptamers of high affinity and specificity. Therefore, the success of the LOC platforms for aptamer-based biosensing will be dependent on the discovery of new and well-functioning aptamers for the target analyte. As aptamers are generally considered reusable target receptors due to their reversible binding and releasing behavior, a crucial component in the LOC system is the washing step to remove the bound analyte molecules from the aptamers to recalibrate the device for the next measurement. Therefore, having a system that efficiently executes the washing procedures that are reliable and reproducible will greatly enhance the reusability of the LOC-based aptasensors. Moreover, an effective and fast target-removal process will be beneficial to the aptamer-based biosensors in achieving continuous and potentially real-time detection of analyte through the automated process on a chip.

## Figures and Tables

**Table 1:** Summary of the aptamer-based microfluidic biosensors.

Detection Principle		Target	Matrix Sample	LOD/ Linear Range	Channel Material	Device Features	Ref.
Electrochemical	Amperometry	Vasopressin	Sheep serum	43 pM	PDMS	Change in CNT conductance.	[40]
	Voltammetry (DPV)	Norovirus	Bovine blood	100 pM 100pM–3.5 nM	PDMS	All-PDMS microfluidic chip. Disposable screen-printed carbon electrode used.	[43]
	Voltammetry (SWV)	Cortisol	Human serum	10 pg/mL, 30 pg/mL–10 µg/mL	SU-8, Quartz	Sample volume (<1 µL). Washing steps not required.	[44]
	Voltammetry (SWV)	Transforming growth factor-beta 1 (TGF-β1)	Human hepatic stellate cell	1 ppb	PDMS	Reconfigurable device prevents electrode fouling.	[46]
	Impedimetric	Creatine kinase-muscle/brain (CK-MB)	Human embryonic stem cell-derived cardiomyocytes	10 pg/mL–100 ng/mL	PDMS	Heart-on-a-chip cardiac bioreactor formed.	[49]
	Impedimetric	A549 human lung carcinoma cell line	Buffer	1.5 × 10^4^ cells/mL, 1 × 10^5^—5 × 10^5^ cells/mL	PDMS	Coplanar 2-electrode configuration used.	[50]
	Impedimetric	A549 human lung carcinoma cell line	Whole blood sample	-	PDMS	Self-assembled monolayer (SAM) of AuNPs forms the detection zone.	[51]
	Impedimetric	H5N1 Avian influenza virus	Buffer	0.0128 HAU (hemagglutinin units)	PDMS	Interdigital gold microelectrode formed.	[52]
	Impedimetric	CCRF-CEM and Ramos cells	T-cell acute lymphoblastic leukemia (ALL)	-	PDMS	Simple detection with digital multimeter.	[53]
FET-based		arginine vasopressin (AVP)	Buffer	1 pM *	PDMS	On-chip resistive microheater and temperature sensor for temperature control.	[67]
		H5N1 Avian influenza virus	Buffer	5 pM	PDMS	Applicable in flow-through sensing.	[69]
Optical	Fluorescence	Ara h 1	Homogenized Biscuit sample	56 ng/mL	PDMS	Capillary-driven retarding valve helps avoid air capture in the microchannel.	[74]
	Fluorescence	Lysozyme, Okadaic acid, Brevetoxin, ß-conglutin lupine	Fresh egg white, Mussel tissue, Sausage	Lysozyme (343 ppb); OA (0.4 ppb); Brevetoxin (0.56 ppb); *ß*-cl (2.5 ppb)	PDMS/ paper	Porous paper avoids complicated surface modification. Comparable with ELISA.	[75]
	Fluorescence	Norovirus	Spiked mussel sample	MWCNT: 4.4 ng/mL GO: 3.3 ng/mL 13 ng/mL to 13 ng/mL	Paper	Works for both 1D (MWCNT) and 2D (GO) carbon nanomaterials.	[76]
	Fluorescence	MCF-7, HL-60, and K562	Cell culture	MCF-7:62 cells/mL, HL-60:70 cells/mL, K562: 65 cells/mL	Paper	Different colored QDs enabled naked eye detection.	[79]
	Fluorescence	Thrombin, prostate specific antigen (PSA), hemagglutinin (HA)	-	-	PDMS	Enables molecular detection on solid surface.	[80]
	Fluorescence	E-Coli	Buffer	Single cells	PDMS	Enables fast and continuous real-time detection.	[81]
	Fluorescence	Influenza A H1N1 virus	-	0.032 HAU	PDMS	Two-aptamer microfluidic system improves sensitivity. Micropump with normally-closed valves enabled efficient transportation of reagents and samples.	[82]
	Fluorescence	Glycated hemoglobins (HbA1c) & Total hemoglobin (Hb)	Blood	-	PDMS	Reagent consumption and analysis time reduced by 75% and 86%, respectively.	[83]
	Fluorescence	Carcinoembryonic antigen (CEA)	Human serum	68 pg/mL 130 pg/mL–8 ng/mL	Glass/ PDMS	Microchip electrophoresis (MCE).	[85]
	Fluorescence	Kanamycin (Kana), Oxytetracycline (OTC)	Milk samples	Kana: 0.001 ng/mL–10 ng/mL OTC: 0.7 pg/mL–0.9 pg/mL	Synthetic Quartz	300-fold signal amplification compared to non-amplified system.	[87]
	Fluorescence	Kanamycin (Kana)	Milk and fish samples	0.3 pg/mL, 0.8 pg/mL—10 ng/mL	-	Reduces matrix interference using ratiometric strategy.	[88]
	Colorimetry	Adenosine	Human serum	1.5 µM 1.5 µM–19.3 mM	Paper	Naked-eye detection.	[90]
	Colorimetry	Cocaine, adenosine, Pb^2+^	Urine	-	Paper	Naked-eye detection.	[91]
	Colorimetry	Thrombin	-	20 pM	PDMS	Naked-eye and Flatband scanner. AgNPs were used.	[89]
	Colorimetry	Thrombin	Human blood	0.083 pg/mL 0.1–50,000 pg/mL	PDMS	Rolling circle amplification (RCA) used.	[95]
	Colorimetry	PfLDH enzyme (Malaria)	Human blood serum	0.01%	CLEAR resin	Smart phone/tablet detection Telemedicine application 3D printing system	[96]
	Chemiluminescence	Glycated hemoglobin (HbA1c).	Blood	0.65 g/dL	PDMS	Aptamer-antibody sandwich assay. Detection time within 25 min.	[99]
	Chemiluminescence	Lysozyme	Human serum	44.6 fM	PDMS	Droplet microfluidics. Very low sample volume of 1 µL.	[100]
	Electrochemiluminescence	Mucin-1	Human serum	8.33 pM 25 pM–50 nM	Paper	3D origami µPAD. Hybridization chain reaction.	[101]
	Electrochemiluminescence	VEGF-165 protein	DMEM cell media	0.17 pM 0.52—52.00 pM	PDMS	Highly selective. In the presence of Ir (III), no signal.	[102]
	Optical interferometry	Plant hormone abscisic acid (ABA)	Plant tissue	0.1 µM	SU8/ PDMS	Capillary microfluidics. No external pumps required.	[104]
	Surface plasmon resonance	Interferon gamma (IFN-γ)	Human plasma	10 pM	Paper	Membrane-based microfluidic disposable device.	[120]
Mass-based	Surface acoustic wave	Prostate specific antigen (PSA) ATP	-	10 ng/mL 10 ng/mL—1 µg/mL	PDMS	Interdigitated transducer.	[126]
	Surface acoustic wave	Thrombin	buffer	-	PDMS	Acoustic wave driven. Interdigitated transducer.	[127]

* In all cases, aptamers were used as the target receptors.
